# Identification of copy number variations in the genome of Dairy Gir cattle

**DOI:** 10.1371/journal.pone.0284085

**Published:** 2023-04-10

**Authors:** Larissa G. Braga, Tatiane C. S. Chud, Rafael N. Watanabe, Rodrigo P. Savegnago, Thomaz M. Sena, Adriana S. do Carmo, Marco A. Machado, João Cláudio do C. Panetto, Marcos Vinicius G. B. da Silva, Danísio P. Munari

**Affiliations:** 1 Departamento de Engenharia e Ciências Exatas, Universidade Estadual Paulista, Jaboticabal, São Paulo, Brazil; 2 Centre for Genetic Improvement of Livestock, Department of Animal Biosciences, University of Guelph, Guelph, Ontario, Canada; 3 Department of Animal Science, Michigan State University, East Lansing, Michigan, United States of America; 4 Departamento de Zootecnia, Universidade Federal de Goiás, Goiânia, Goiás, Brazil; 5 Embrapa Gado de Leite, Juiz de Fora, Minas Gerais, Brazil; Mohammed VI Polytechnic University, MOROCCO

## Abstract

Studying structural variants that can control complex traits is relevant for dairy cattle production, especially for animals that are tolerant to breeding conditions in the tropics, such as the Dairy Gir cattle. This study identified and characterized high confidence copy number variation regions (CNVR) in the Gir breed genome. A total of 38 animals were whole-genome sequenced, and 566 individuals were genotyped with a high-density SNP panel, among which 36 animals had both sequencing and SNP genotyping data available. Two sets of high confidence CNVR were established: one based on common CNV identified in the studied population (CNVR_POP), and another with CNV identified in sires with both sequence and SNP genotyping data available (CNVR_ANI). We found 10 CNVR_POP and 45 CNVR_ANI, which covered 1.05 Mb and 4.4 Mb of the bovine genome, respectively. Merging these CNV sets for functional analysis resulted in 48 unique high confidence CNVR. The overlapping genes were previously related to embryonic mortality, environmental adaptation, evolutionary process, immune response, longevity, mammary gland, resistance to gastrointestinal parasites, and stimuli recognition, among others. Our results contribute to a better understanding of the Gir breed genome. Moreover, the CNV identified in this study can potentially affect genes related to complex traits, such as production, health, and reproduction.

## Introduction

Dairy Gir animals are tolerant to heat stress, diseases, and tropical parasites [1], making them a relevant genetic resource for milk production in the tropics. Due to climate change, the Gir breed could become important in temperate regions, mainly in crosses with taurine animals [2]. The National Dairy Gir Breeding Program (PNMGL) uses DNA information to identify genetic variants of beta-casein and perform a genomic selection of bulls and cows. Therefore, identifying structural DNA variants influencing quantitative traits, such as copy number variations (CNV), is essential for Dairy Gir cattle genetic improvement. CNV involves the presence of deletions and duplications greater than 50 base pairs (bp) between two individuals of a species [3]. CNV can functionally contribute to the processes of domestication, breed formation [4, 5], differentiation between indicine and taurine cattle [6, 7], and environmental adaptation [7, 8], and it may also provide adaptive advantages to individuals [6, 9]. In previous studies with cattle, CNV regions (CNVR) were related to milk production [10], hoof health traits [11] and residual feed intake [12] in Holstein cattle, stature in Chinese cattle breeds [13], navel length in Zebu cattle [6], and calf mortality in Wagyu cattle [14].

The CNV identified from whole-genome sequencing (WGS) and single nucleotide polymorphism (SNP) genotyping panels can vary in number, length, and distribution in the genome [15, 16]. CNV detection using SNP panels is essentially based on two measurements: Log R Ratio (LRR) and B allele frequency (BAF) from the genotyping process [17]. In WGS data, structural variants (SV) are predicted from abnormal alignment patterns that suggest genomic rearrangement breakpoints. There are four main approaches: read-pair (RP), split-read (SR), read-depth (RD), and assembly-based (AS). Although WGS data approaches are generally considered more precise and accurate, they may also call false positive variants [18–20]. An alternative strategy to improve the probability of reliable CNV detection is incorporating different detection approaches and molecular techniques, such as WGS and SNP panels.

Despite CNVR ranging from two up to 7% of the bovine genome [21], genomic selection in this species has been only directed toward using SNP markers. Genomic prediction integrating SNP and CNV can offer new insights to elucidate complex traits and understand the proportion of genetic variation not explained by SNP (missing heritability) [22]. The same study reported that the genomic prediction integrating SNP and common deletions—present in at least 5% of the population—resulted in increased accuracy for some traits in Nelore cattle.

The first step toward including SV, such as CNV, in genomic predictions and genome-wide association studies (GWAS) is detecting and mapping this type of genomic variant. Thus, the objectives of this study were to: (1) detect CNV in Dairy Gir cattle, (2) define high confidence CNVR using two in silico methods, and (3) determine the genomic regions where high confidence CNVR occurs that coincide with genes and quantitative trait loci (QTL) previously related to production traits.

## Material and methods

### Samples, alignment, and preparation of sequencing data

Sires from PNMGL, conducted in partnership with the Brazilian Association of Dairy Gir Breeders (ABCGIL) and Embrapa Dairy Cattle, were ranked based on a study of their performance and progeny number in the PNMGL. The sires that had the most progeny in PNMGL and were representative of all lineages in the population were selected for WGS. Genomic DNA was extracted from semen straws obtained from commercial artificial insemination centers in Brazil. The institutional research ethics board of the São Paulo State University and EMBRAPA did not require ethics approval for this study.

The DNA extraction and WGS were divided into two sets. For samples one to 13, DNA extraction was performed using the DNeasy Blood & Tissue Kit (Qiagen, Valencia, CA, USA), according to the manufacturer’s recommendations. The extracted DNA was quantified and evaluated by the NanoDrop 1000 spectrophotometer (Thermo Scientific, Wilmington, DE, USA). The samples were sequenced using the Illumina HiSeq2000 (Illumina Inc., San Diego, CA, USA). The paired-end sequencing produced 2 x 100 bp and 2 x 200 bp reads, with an average sequencing coverage of 13.9X.

For samples 14 to 38, DNA was extracted using a saline buffer and phenol/chloroform purification protocol, briefly described by Machado et al. [23]. The concentration and quality of the isolated DNA were quantified using the Qubit fluorometer 2.0 (Life Technologies, Grand Island, NY). The Illumina TruSeq Nano kit (Illumina Inc., San Diego, CA, USA) was used for library preparation according to the protocols recommended by the manufacturer. The samples were sequenced using the Illumina NovaSeq 6000 (Illumina Inc., San Diego, CA, USA). Reads measuring 2 x 150 bp were produced, with average sequencing coverage of 16.7X per sample.

The quality of the reads was evaluated using the FastQC tool (v. 0.11.8) (http://www.bioinformatics.babraham.ac.uk/projects/fastqc/), and the quality control followed the parameters recommended by the 1000 Bull Genomes Project protocol (http://www.1000bullgenomes.com, last accessed on 11/20/2020). The SeqyClean software [24] was used to remove: (1) reads with three or more unidentified bases (N) in the sequences, (2) reads with an average quality score less than or equal to 20 for Phred score (meaning a maximum probability that the bases are incorrect of 0.01), and (3) reads less than 50 bases in length. Additionally, adapter sequences and possible contaminants were also removed.

The reads from both sets were aligned to the bovine reference genome ARS-UCD 1.2 using the mem option of the BWA algorithm [25] (v. 0.7.15-r1144-dirty). Conversion to binary format, sorting, and indexing were completed by Samtools [26] (v. 1.8), using the options view, sort, and index, respectively. Optical and PCR duplicates were removed by the MarkDuplicates option of Picard Tools [27] (v. 2.18.2-SNAPSHOT). Base quality score recalibration (BQSR) was done using BaseRecalibrator and PrintReads of the Genome Analysis Toolkit [28] (GATK, v. 3.8-1-0-gf15c1c3ef). BQSR is a data processing step that identifies systematic errors generated by the sequencing machine. All these steps followed the parameter recommendations by the 1000 Bull Genomes Project guidelines. The set of known variants provided by the 1000 Bull Genomes project consortium was applied for BQSR. The flagstat option of Samtools was used to calculate alignment statistics.

### Genotyping samples

Sampling was conducted by herders during routine husbandry practices in their commercial herds without research purposes. The institutional research ethics board of the São Paulo State University and EMBRAPA did not require ethics approval for this study. Samples from 566 Dairy Gir animals were genotyped using the Illumina BovineHD BeadChip panel (Illumina Inc., San Diego, CA, USA), which consists of 777,962 SNP distributed along the genome, with a mean distance between markers equal to 3.43 kilobases (Kb) and median equal to 2.68 Kb. SNP with a GenCall score below 0.15 were removed for quality control [29].

Out of the 566 genotyped animals, 36 individuals were also whole-genome sequenced. Principal component analysis (PCA) was conducted using the genotype matrix to verify if there was any population structure among the animals and evaluate the representativeness of the sequenced individuals. The SNP map used was based on the reference genome ARS-UCD1.2, and only autosomes SNP with known positions in the ARS-UCD1.2 assembly (720,731 markers) were used in the analysis. For PCA, SNP with minor allele frequency (—maf) less than 5% and call rate less than 90%(—geno), and samples with a call rate less than 90% were removed (—mind) using the PLINK software [30] (v.1.9).

### CNV detection from sequencing data

CNVnator [31] (v. 0.4.1) was used for CNV detection. This software uses the RD approach and performs a correction for the genome’s guanine-cytosine (GC) content. While RD is one of the most common approaches for CNV detection, it is less robust for the accuracy of the CNV breakpoints resolution [19].

CNV detection was carried out only in autosomal chromosomes with a bin size of 250 bp and a mean RD signal of 4.12, which aligns with Abyzov et al. recommendations [31]. Only CNV larger than 1 Kb and smaller than 5 Mb [22], significant (p<0.05) for the t-statistic test, in which the null hypothesis is if the mean signal of reads depth in the CNV region is the same as the average signal depth in the sample, and with a fraction of low-quality mapped reads (q_0_) less than 0.5 were considered for analysis.

DELLY [32] (v. 0.7.6) was also used for CNV detection to increase reliability. This software applies the RP and SR approaches to detect CNV. The RP algorithm analyzes read libraries for discordantly mapped read pairs. Then, the SR approach is used to refine the definition of the SV breakpoints predicted by the RP approach.

DELLY enables the detection of duplication and deletion events in all individuals simultaneously since CNV identified in one individual (singletons CNV) tends to be false positives compared to CNV identified in several individuals [33]. CNV detection was performed only in autosomal chromosomes. The minimum mapping quality option (-q), which is the probability that a read is aligned in the wrong place, was set to a value of 20, following the criteria of Khan et al. [32]. Only CNV larger than 1 Kb and smaller than 5 Mb [22] and CNV with support from more than four read pairs (paired-end support) were considered [34].

### CNV identified from SNP genotyping

CNV detection using SNP panels was performed by PennCNV [17] (v. 1.0.5). This software applies Bayesian methodologies of the hidden Markov model utilizing the Log R Ratio (LRR) to measure the total signal intensity and the B Allele Frequency (BAF) to measure the proportion of the B allele in the sample. The population frequency of B allele information was calculated using the BAF value of each SNP in all samples.

To reduce false-positive results, the LRR values of each SNP were adjusted for the genomic waves along the genomic regions, taking into account the expected GC content in the bovine genome in a region of 500 Kb around each SNP. Genomic waves refer to a signal noise related to the GC content in the genome, which interferes with accurate CNV detection. Genomic waves are defined as a genome-wide spatial autocorrelation or ‘wave’ pattern in signal intensity data across all chromosomes [35]. Pedigree information was not used in the CNV detection. CNV with more than 10 SNP, an LRR standard deviation no more than 0.30, BAF drift no more than 0.01, a waviness factor no more than 0.05 [16], and CNV ranging from 1 Kb to 5 Mb in length were maintained [22].

### High confidence CNVR

The CNVR identified from the results of different molecular techniques can be considered as having high confidence [15]. Two sets of high confidence CNVR (CNVR_POP and CNVR_ANI) were established to increase results reliability [16]. The CNVR_POP contained common CNVR identified in all the studied population, and the CNVR_ANI included CNV identified in the representative animals with both WGS and SNP genotyping data available.

To define the CNVR_POP set, CNVR detected from WGS data (CNVR_SEQ) and SNP genotyping (CNVR_GEN) were used. The CNVR were determined by grouping CNV that overlapped by at least 1 bp within each algorithm, using the merge option of the Bedtools program [36] (v. 2.26). In CNVR_SEQ, a minimum reciprocal overlap criterion of 50% was considered between the CNVR detected by DELLY and CNVnator software, using the Intersect option of Bedtools. After that, overlapping CNVR between CNVR_GEN and CNVR_SEQ sets were selected, with the same minimum reciprocal overlap criterion. From this, only the CNVR present in at least 5% of the population were selected for the CNVR_POP set (S1 Fig).

To establish the CNVR_ANI set, only the 36 individuals that were both whole-genome sequenced and genotyped were considered. For each of these individuals, common CNV identified from the SNP panel and WGS data that reciprocally overlapped at least 50% were retained. CNVR were determined by grouping CNV overlapping at least 1 bp. Only the results of the PennCNV and CNVnator software were used in CNVR_ANI, as the detection of CNV was performed by sample (S1 Fig).

The CNVR_POP and CNVR_ANI sets were merged for further analysis. Samplot software [37] was used to visualize the unique high confidence CNVR identified in the WGS data.

### Functional analysis

Genes and QTL were retrieved from the Ensembl Genes database (Ensembl Release 104) (https://www.ensembl.org/, last accessed 05/11/2021) and the Animal Genome database (https://www.animalgenome.org/cgi-bin/QTLdb/BT/index, last accessed 05/11/2021), respectively. The GALLO package [38] from R software [39] was used to identify genes and QTL overlapping unique high confidence CNVR. Terms from the Gene Ontology (GO) database and biological pathways predicted by the Kyoto Encyclopedia of Genes and Genomes (KEGG) database (https://www.genome.jp/kegg/, last accessed on 06/10/2021) were enriched (FDR< 0.05) using WebGestaltR package [40] in R software [39]. The enrichment analysis was performed using the hypergeometric Over-Representation Analysis test. Biological terms in the Gene Ontology are divided into three groups: Cellular Components, Biological Processes, and Molecular Functions.

Terms from the Medical Subject Headings (MeSH) (https://www.ncbi.nlm.nih.gov/mesh) were used for gene enrichment analysis (p-adjusted<0.05) through the meshes package [41] in R software [39], using the gene2pubmed database option. The MeSH terms = were Anatomy (A), Disease (C), Drugs and Chemicals (D), and Biological Sciences (G). The information about the overlapping genes was obtained from RefSeq Genes (https://www.ncbi.nlm.nih.gov/refseq/rsg/, last accessed on 06/10/2021) and GeneCards (https://www.genecards.org/, last accessed on 06/10/2021).

### Comparison of CNVR with previous studies

To compare CNVR from previous studies, autosomal CNV from eight studies available on the Genomic Variant archive database (DGVa) of EMBL-EBI (https://www.ebi.ac.uk/dgva, last accessed on 10/15/2021) were compared to the unique high confidence CNVR identified in this study. Only two studies included samples of Gir animals [4, 42], and three included other Zebu breeds [4, 42, 43]. One study detected CNV using array comparative genomic hybridization (array CGH) [4], three studies used SNP panel data [42–44], and four studies used WGS data [9, 45–47]. The number of breeds in the studies varied from one to 21, and the sample size ranged from six to 539. To form the DGVa CNVR set, chromosome, start position, end position, type, and study information were retrieved.

Copy number variants in those articles were detected using both bovine reference genomes UMD3.1 [48] and BTAU_4.0 [49]. The variant’s coordinates were translated to ARS-UCD1.2 using the UCSC Genome Browser LiftOver tool [50]. The minimum ratio of bases that had to be remapped was set to 0.4 [16], and default values were used for all other LiftOver parameters. After translation to ARS-UCD1.2 positions, CNV overlapping at least 1 bp were merged. The DGVa CNVR set resulted in 8,797 CNVR. The unique high confidence CNVR and the DGVa CNVR were considered equal if the reciprocal overlap between them was at least 50%.

## Results

### Alignment and pre-processing of sequencing data

After removing duplicates, paired-end sequencing produced 13,530,707,923 reads, where the average total number of reads was 356,071,261 (min: 245,377,907, max: 486,209,902, median: 363,454,380, standard deviation–SD: 50,991,373). On average, 99.58% were mapped (min: 96.59%, max: 99.88%, median: 99.79%, SD: 0.69%). The average number of properly paired reads was 94.88% (min: 84.38%, max: 97.98, median: 96.06%, SD: 3.63%). The mean coverage was 16.36X (min: 10.20X, max: 25.00X, median: 15.95X, SD: 2.99X) (S1 Table).

### CNV identified from sequencing data

For the CNVnator software, an average of 2,143 CNV per animal were detected (min: 1,554, max: 3,844, median: 1,940, SD: 564.93). The total number of CNV was 81,447, with 53,876 deletions and 27,571 duplications. The mean size of the CNV was 17,239 bp (min: 1,249 bp, max: 1,791,499 bp, median: 7,999 bp, SD: 42,662.99 bp). Pearson’s simple linear correlation between CNV number and coverage was positive and significant (0.34, p = 0.04); this result was expected due to the RD approach.

For the DELLY software, CNV detected in more than one individual were considered populational CNV, and those detected in only one animal were singletons CNV. Multiple detections of 38 individuals generated 20,888 variants (20,351 populational CNV and 537 singletons CNV). A total of 14,571 deletions (14,186 populational CNV and 385 singletons) and 6,317 duplications (6,165 populational and 152 singletons) were detected. The mean size of the CNV was 179,007 bp (min: 1,000 bp, max: 4,983,990 bp, median: 11,518 bp, SD: 551,161.4 bp).

### Genotyping samples

**Fig 1:**
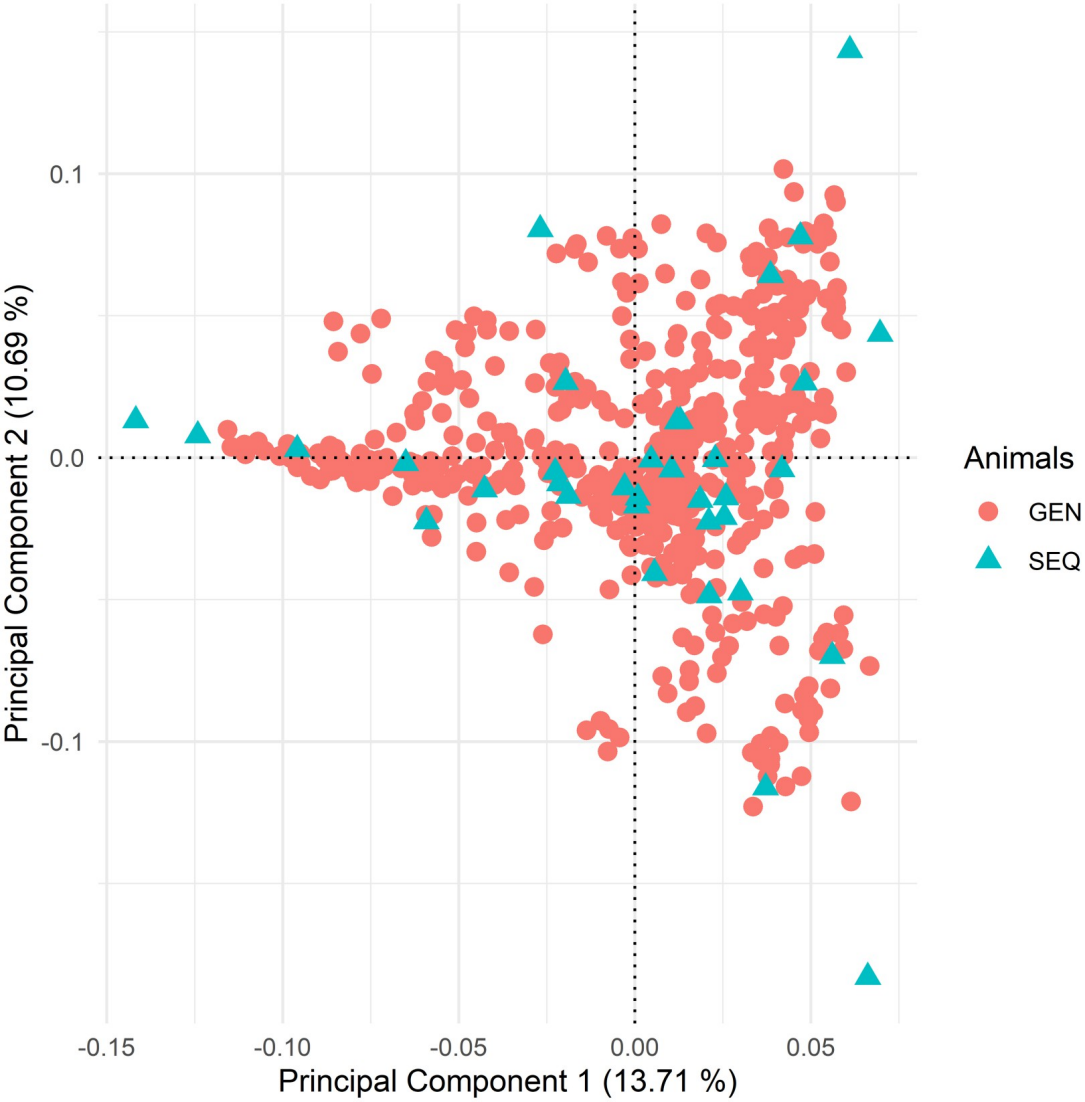
Principal component analysis of individuals genotyped with a high-density SNP panel. This figure shows the animals that were only genotyped (GEN) and those that were also sequenced (SEQ).

The mean SNP value per animal for the genotyped animals was 770,125 (min: 666,135, max: 774,163, median: 772,024, SD: 10,090.94). After quality control, 433,015 SNP remained, and five animals were removed. No stratification was observed in the population. The genotyped and sequenced animals were randomly distributed on the two-dimensional plot, representing the diversity of genetic distances within the genotyped population (Fig 1).

### CNV detected from SNP genotyping

The SNP map was based on the reference genome ARS-UCD1.2, consisting only of autosomal SNP with known positions in the ARS-UCD1.2 assembly (720,731 SNP). In the SNP map, 7.35% of SNP were removed because they were not positioned on autosomal chromosomes or had no known position in the ARS-UCD1.2 reference genome, and 9.46% of SNP were not used due to their low GenCall score in the population.

After quality control, 547 animals and 652,560 SNP were used for PennCNV detection. A total of 4,162 CNV were identified, with 2,510 deletions and 1,650 duplications. The mean number of CNV per animal was 7.6 (min: 1, max: 90, median: 7, SD: 7). The mean number of markers in each CNV was 25.16 (min: 10, max: 293, median: 17, SD: 19.26). The mean size of the CNV was 122,807 bp (min: 10,180 bp, max: 1,371,933 bp, median: 58,988 bp, SD: 120,392.8 bp).

### High confidence CNVR

In the CNVR_GEN, 489 CNVR were detected, with a mean size of 95,170 bp (min: 10,714 bp, max: 1,410,517 bp, median: 50,517 bp, SD: 127,725.6 bp), covering a total of 46,538,246 bp of the genome. Among them, 428 were deletions, 55 were duplications, and six were considered complex, where both duplications and deletions occurred.

**Fig 2:**
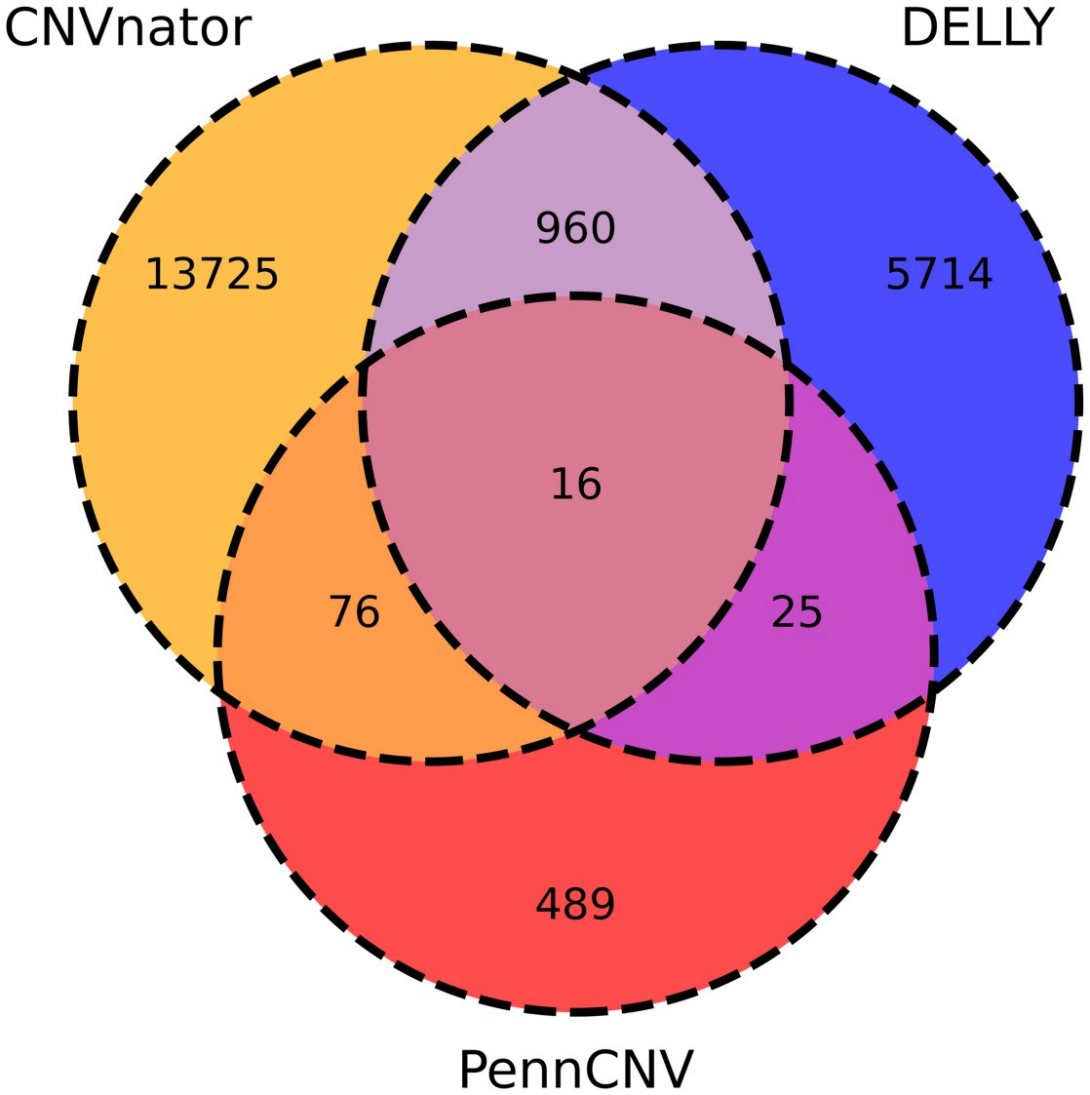
Copy number variation regions identified from whole-genome sequencing data using CNVnator and DELLY software and from SNP panel data using PennCNV software.

Using WGS data, CNVnator detected 13,725 CNVR, out of which 7,204 were deletions, 4,961 duplications, and 1,560 complexes. The mean size was 34,080 bp (min:1,249 bp, max: 2,772,749 bp, median: 10,999 bp, SD: 90,257.1 bp). On the other hand, DELLY software identified 5,714 CNVR, of which 4,003 were deletions, 443 were duplications, and 1,268 were complexes, with a mean size of 194,892 bp (min: 1,001 bp, max: 12,426,687 bp, median: 10,999 bp, SD: 821,053.5 bp). Fig 2 presents the number of CNVR identified by each software. In the CNVR_SEQ set, 960 CNVR were identified, with an average size of 22,786 bp (min: 1,111 bp, max: 2,006,399 bp, median: 3,346 bp, SD: 104,755.6 bp), covering 21,874,126 bp, of these 728 were deletions, 63 were duplications, and 169 were complex CNVR.

Regarding the CNVR_POP set, ten CNVR were found in eight chromosomes, with an average size of 104,943 bp (min: 14,879 bp, max: 521,437 bp, median: 52,933 bp, SD: 151,104.4 bp), covering 1,049,430 bp. Among these, four were deletions, two were duplications, and four were complex CNVR (S2 Table). Four CNVR were present in more than 10% of the population, and one was present in more than 30%. The CNVR from CNVR_POP were detected in 25 sequenced animals.

For the CNVR_ANI set, 240 CNV were detected using SNP panel data, and 77,582 were detected using WGS data. After overlapping the CNV from both data sets, 45 CNVR were identified in 21 chromosomes, with a mean size of 97,931 bp (min: 12,003 bp, max: 355,151 bp, median: 53,140 bp, SD: 96,949, 66 bp), covering 4,406,887 bp. Among them, 23 were deletions, and 22 were duplications (S3 Table).

**Fig 3:**
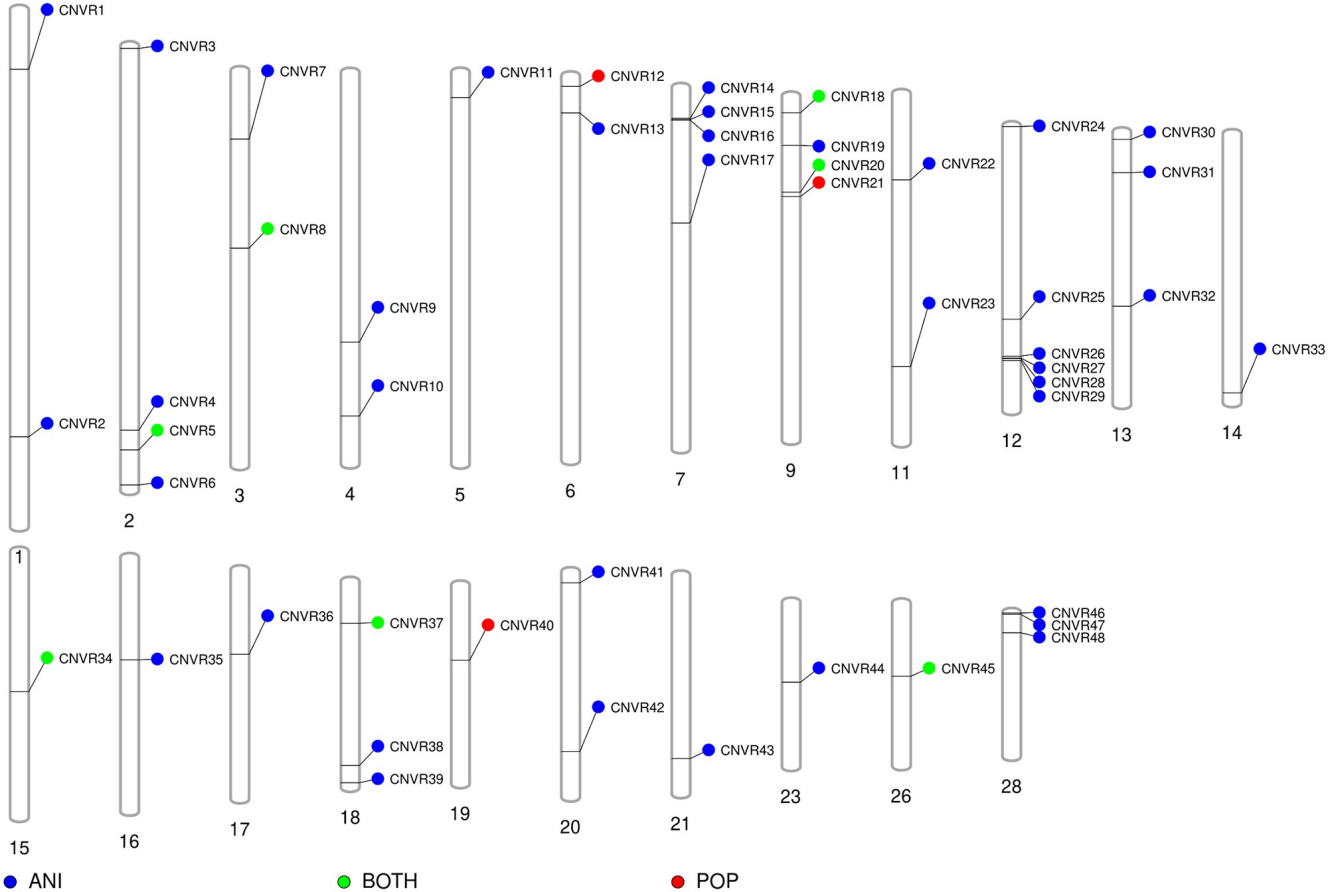
Distribution of unique high confidence copy number variation regions (CNVR) in the bovine genome. The CNVR_ANI (ANI), CNVR_POP (POP), and CNVR sets present in both sets (BOTH) are represented. Only autosomal chromosomes with CNVR are represented.

Finally, after overlapping CNV from CNVR_POP and CNVR_ANI sets, 48 unique high confidence CNVR were retained for functional analysis (Fig 3) (S4 Table). Out of these, seven CNVR (70% of the CNVR_POP set) were shared between CNVR_POP and CNVR_ANI.

### Functional analysis

According to RefSeq Genes and Gene Cards, 69 genes and two pseudogenes were annotated in 31 unique high confidence CNVR (64.58%) (S5 Table). Among these, 21 genes and two pseudogenes from the olfactory receptor family (ex: *OR2L13*, *OR2L2*, *OR1P1*) overlapped with CNVR14 (BTA7: 9455783–9693750), CNVR16 (BTA7:10055082–10135500), CNVR17 (BTA7:41582849–41938000), CNVR34 (BTA15:44870278–44942116), CNVR40 (BTA19:23956716–23987626), CNVR46 (BTA28:123251–413750) (S2–S7 Figs). Three guanylate binding proteins (GBP) genes (*GBP2*, *GBP4*, *GBP6*) were found in CNVR8 (BTA3:54329751–54851188) (S8 and S9 Figs). GBP participates in innate immunity against several intracellular pathogens [51]. Another six immunity-related genes (*HERC2*, *CLEC5A*, *SIRPB1*, *BANP*, *BoLA-DQB*, *BoLA-DQA1*) overlapped with CNVR3 (BTA2:719378–745361), CNVR10 (BTA4:105218001–105292500), CNVR32 (BTA13:534618–53511604), CNVR37 (BTA18:13328574–13397500), and CNVR44 (BTA23:25679501–25705975) (S10–S14 Figs). The CNVR overlapped with exonic regions in all genes and pseudogenes.

In 14 unique high confidence CNVR (29.17%), 156 QTL were found, of which 44 QTL were significantly associated (p<0.05) with production traits (29.54%), reproduction (22.73%), conformation (18.18%), health (13.64%), milk (13.63%), and meat and carcass (2.27%) (S5 Table). Most QTL (52.27%) overlapped regions where only duplication events occurred, 43.18% QTL overlapped regions where deletion events occurred, and 4.54% overlapped complex regions.

In the enrichment analysis of significant GO terms (FDR<0.05), the term ‘stimulus detection’ (GO:0051606) was observed in the Biological Processes category, and the term ‘olfactory receptor activity’ (GO:0004984) was observed in the Molecular Functions category. These two terms were related to five genes (*OR1P1*, *OR5D18K*, *OR2L13*, *OR2T22*, *OR2M16*). No significantly enriched terms (FDR>0.05) were found for the Cell Components category. No significant enriched biological pathway predicted by the KEGG database (FDR>0.05) was observed.

In the enrichment analysis of significant MeSH terms (p-adjusted<0.05), the term ‘CD4+ T lymphocytes’ was found in the Anatomy category, three terms (‘Antigen Presentation’, ‘Genes, Duplicate’, ‘DNA Copy Number Variations’) in the Biological Sciences category, and 13 in the Chemicals and Drugs category. These terms were related to at least one of the *BoLA-DQB* (also known as *DQB1*), *BoLA-DQA1* (also known as *LOC100848815*), and *GBP4* genes (S6 Table). No significantly enriched terms (p-adjusted>0.05) were found in the Disease category.

### Comparison of CNVR with previous studies

Comparing the unique high confidence CNVR identified in this study with DGVa CNVR set showed only a few overlapping CNVR. The eight overlapping CNVR are listed in S7 Table. Each DGVa study was represented by at least two overlapping regions. Our study identified seven unique high confidence CNVR that overlapped with the CNV detected by Mesbah-Uddin et al. [47].

## Discussion

A total of 547 animals were used in this study, of which 36 had both WGS and SNP genotyping data available. CNV were called using both data sources and different detection approaches. Two *in silico* molecular techniques were used to identify high confidence CNVR related to the individuals and population studied, resulting in 45 and ten high confidence CNVR, covering 4.4 Mb and 1.05 Mb, respectively. The functional analysis of the regions covered by CNVR revealed genes related to complex traits.

Although the CNV were identified from the same animals, WGS data resulted in 325 times more CNV than SNP panels. Similarly, Butty et al. [16] and Zhan et al. [15] also found differences in the number of CNV detected between the SNP panel and WGS data in cattle. These molecular techniques differ in their coverage range and capabilities for detecting and solving CNV breakpoints [15]. Certain CNV detected only from WGS data may represent true variants. However, they are unlikely or impossible to be detected by high-density SNP panels [52] due to quantity, distribution [17], and the pre-established position of markers [53]. Furthermore, multiple and adjacent CNV could result in overestimating the CNV size in SNP panel-based algorithms [42].

The CNVR_ANI set was defined to detect high confidence CNVR present in representative bulls. CNVR_ANI set was obtained by verifying the CNV found using SNP panels data and RD approach in the WGS data. Both algorithms rely on similar information where the amount of DNA present in a given region is indirectly used to identify CNV in each sample [16]. In the RD approach, this is indirectly measured by the coverage of each segment [31]. In SNP panels, the fluorescence signal intensity for each probe at the time of genotyping also reflects the amount of DNA in a given position [17].

The CNVR_POP set can be considered as copy number polymorphisms, as they are present in more than 1% of the studied population. Additionally, the methodology used to identify CNVR_POP can be used as a criterion for selecting CNV to be validated by qPCR (real-time PCR) in future studies of the Dairy Gir population. FISH (Fluorescent in Situ Hybridization) and qPCR are widely accepted methods for validating CNV, as they provide high accuracy and specificity [9]. However, these analyses are known to be time-consuming, expensive, and require a significant amount of biological material. In light of these limitations, this study chose to focus on an in-silico approach as a way to identify high confidence CNVR while minimizing the need for extensive laboratory resources [9].

The strategy to establish high confidence CNVR sets (CNVR_POP and CNVR_ANI) may have reduced the number of CNVR. However, the focus of this study was quality in detection, as CNV can be partially validated when the same region containing copy number variants is detected using WGS and SNP panel data [16]. Due to the false-positive calls inherent in CNV detection approaches and the limitations of experimental validation in a large number of animals, the combination of different molecular techniques can provide SV identification with high confidence [15]. Additionally, up to 48% of PennCNV calls are likely false positives [54]. Thus, partial validation using WGS data is an alternative to improve CNV detection reliability.

Using information from SNP panels and analyzing WGS data with more than one approach may increase the accuracy of CNV detection [15]. Integrating RD, SR, and PE approaches can decrease the false positive rate during CNV detection compared to using a single algorithm [7, 18]. The main weakness of the RD approach is its limited ability to identify the breakpoints accurately. However, this limitation can be addressed by incorporating RP and SR approaches [19, 20]. Despite this, the choice of algorithm plays a crucial role in the overall reliability of the combinatorial methodology. Regardless of the WGS approach used, overlapping SV call with high precision and high recall to select pairs of algorithms will directly impact the accuracy of the results, irrespective of the combinations of methods utilized by the algorithms [18].

The accuracy of CNV detection and the definition of their boundary can be highly increased with long-read sequencing [55]. However, the high cost may limit its usage on a large scale. This supports our decision to apply the three approaches for CNV detection in WGS data.

Further analyses are needed to investigate the relationship between CNVR and economically relevant traits. Some genes found in the unique high confidence CNVR were previously related to reproductive and health traits. The CNVR19 (S15 Fig) overlapped with SENP6 (*SUMO Specific Peptidase 6*) and *FILIP1* (*Filamin A Interacting Protein 1*) genes. CNVR present in these two genes were associated with sheep’s litter size [56]. SENP6 is a sumoylation protease that is a critical regulator of aging and skeletal development [57]. The *FILIP1* gene is involved in skeletal muscle cell differentiation [58].

The genes *FILIP1*, *SENP6*, *CA5A* (*Carbonic anhydrase 5A*), and *BANP* (*BTG3 Associated Nuclear Protein*) were related to the longevity trait in Chinese Holstein cattle [59]. The *CA5A* was mapped in CNVR37 (S13 Fig) and was previously reported in selection signature regions, which may be related to environmental adaptation in Iraqi cattle breeds [60]. *CA5A* gene was related to high fertility in Holstein cattle in a co-expression meta-analysis [61]. CA5A protein catalyzes the reversible conversion of CO_2_ to a proton and a bicarbonate ion. *CA5A* activity was reported in the ovary and uterine epithelium [62, 63]. The *BANP* gene was also mapped in CNVR 37. This gene encodes the BANP protein, which activates and regulates the transcription of genes involved in metabolism, DNA damage response, chromatin opening, and chromosomal segregation during mitosis [64, 65].

The CNVR3 (S10 Fig) overlapped with the *HERC2* (*HECT And RLD Domain Containing E3 Ubiquitin Protein Ligase 2*) gene, which was previously related to perinatal mortality in taurine cattle [66] and initial sperm motility in Angus breed [67]. *HERC2* encodes an E3 ubiquitin-protein ligase that targets proteins involved in cell cycle regulation, mitochondrial bioenergetics, and DNA damage response [68]. The *RHOU* gene (*ras homolog family member U*) was mapped in CNVR46 and encodes a protein of the RHO family of GTPases (guanine triphosphatases), which regulates fundamental processes for mammary gland development [69].

In cattle, CNV are highly enriched with immunity and defense genes, indicating that CNV contribute to their large variability [5, 8, 70]. The *guanylate binding protein* (*GBP*) genes *GBP2*, *GBP4*, *and GBP6* were found in CNVR8 (S8 and S9 Figs). GBP are relevant in eliminating intracellular parasites, and this process is mediated by IFN-γ (interferon-γ) during the innate immune response [71]. The *GBP6* gene plays a relevant role in the intracellular killing of *Mycobacterium avium* subspecies *paratuberculosis* in cattle, contributing to the immune response against this pathogen [72]. CNV in the genes of the GBP family (*GBP2*, *GBP4*, *GBP5*, and *GBP7*) were previously associated with residual feed intake in Holstein cows [12]. A complex copy number polymorphism region in the *GBP4* gene was found to be negatively associated with stature in Chinese cattle [13]. Additionally, selection signatures overlapping the genes *GBP2*, *GBP4*, *and GBP6* were found in Swiss cattle breeds adapted to cold climates and high altitudes [73].

Two genes belonging to the major bovine histocompatibility complex (MHC) class II region, *BoLA-DQB* and *BoLA-DQA1*, were found in CNVR44. MeSH terms related to the immune system and gene duplication were enriched in these two genes. These genes overlapped with selection signatures in Nelore cattle, another Zebu breed [74]. Class II molecules are expressed on cells that present antigen epitopes (e.g., dendritic cells) to CD4+ T lymphocytes that, once stimulated, can activate macrophages and B lymphocytes, provoking an inflammatory response and antibody production [75]. *BoLA-DQA1* was associated with the proviral load of the bovine leukemia virus, which causes enzootic bovine leukosis (EBL). The load can be considered a diagnostic index for determining EBL’s progression and transmission risk [76].

Among the overlapped genes, 30.43% belong to the olfactory receptor (OR) family. These genes were found in CNVR14, 17, 18, 34, 40, and 41 (S2, S4–6, S16 and S17 Figs), where deletion or complex events occurred. Also, GO terms ‘stimuli detection’ and ‘olfactory receptor activity’ were enriched. The expression and regulation of OR genes are critical for cattle regarding the reception of information about the environment and communication between animals through pheromone recognition [77]. Olfaction is crucial in various tasks, including avoiding dangers, identifying mates and offspring, and marking territory. The OR gene family is known for its high variability across different vertebrate species, including cattle [78]. This high variability is characterized by frequent CNV events [8, 11, 16, 70], suggesting that evolutionary forces may be at play and that the OR genes are under selective pressure [70, 79]. Genomic variations in olfactory genes, such as SNP and CNV, are associated with stress in humans [80], hoof disorders in Holstein cattle [11], and saturated fatty acid profile in Nelore cattle [81].

Approximately 83% of the unique high confidence CNVR did not overlap with the DGVa CNVR set. The CNV and CNVR found in this study establish a basis for future research on SV in Zebu. Further research should be undertaken to investigate the effect of including CNV information in genomic selection in Dairy Gir cattle. Additionally, CNV-based GWAS studies for critical traits in Dairy Gir cattle are strongly encouraged.

## Conclusions

Our findings detected and characterized 48 high confidence CNVR in the Dairy Gir cattle genome, contributing to a better understanding of the Gir breed genome. These results offer an alternative for selecting CNV to be validated in the population. Furthermore, the identified CNVR have the potential to affect genes involved in the evolutionary process and the phenotypic variation of essential dairy industry traits, such as lactation, fertility, stimuli recognition, and health.

## Supporting information

S1 Fig Definition of the high confidence copy number variation region sets (CNVR). (A) CNVR_POP set. (B) CNVR_ANI set.
(DOCX)

S2 Fig Graphical visualization of CNVR14 (BTA7: 9455783–9693750) across different samples showing putative deletion events. (DOCX)

S3 Fig Graphical visualization of CNVR16 (BTA7:10055082–10135500) across different samples showing putative deletion events. (DOCX)

S4 Fig Graphical visualization of CNVR17 (BTA7:41582849–41938000) across different samples showing putative deletion events. (DOCX)

S5 Fig Graphical visualization of CNVR34 (BTA15:44870278–44942116) across different samples showing putative deletions events. (DOCX)

S6 Fig G Graphical visualization of the CNVR40 (BTA19:23956716–23987626), across different samples showing putative complex events. (DOCX)

S7 Fig Graphical visualization of CNVR46 (BTA28:123251–413750) across different samples showing putative deletion events. (DOCX)

S8 Fig Graphical visualization of CNVR8 (BTA3:54329751–54851188) across different samples showing putative deletion events. (DOCX)

S9 Fig Graphical visualization of CNVR8 (BTA3:54329751–54851188) across different samples showing putative duplication events. (DOCX)

S10 Fig Graphical visualization of CNVR3 (BTA2:719378–745361) across different samples showing putative duplication events. (DOCX)

S11 Fig Graphical visualization of CNVR10 (BTA4:105218001–105292500) across different samples showing putative duplication events. (DOCX)

S12 Fig Graphical visualization of CNVR32 (BTA13:534618–53511604) across different samples showing putative deletion events. (DOCX)

S13 Fig Graphical visualization of CNVR37 (BTA18:13328574–13397500) across different samples showing putative duplication events. (DOCX)

S14 Fig Graphical visualization of CNVR44 (BTA23:25679501–25705975) across different samples showing putative deletion events. (DOCX)

S15 Fig Graphical visualization of CNVR19 (BTA9:15095199–15271750) across different samples showing putative duplication events. (DOCX)

S16 Fig Graphical visualization of CNVR18 (BTA9:5051796–5177690) across different samples showing putative deletion events. (DOCX)

S17 Fig Graphical visualization of CNVR41 (BTA20:3549957–3609244) across different samples showing putative deletion events. (DOCX)

S1 Table Sample, total number of reads, percentage of mapped reads (%), percentage of properly paired reads (%), and coverage (X) per sample after duplicates removal. (DOCX)

S2 Table Chromosome, start and end position, size in base pairs (bp), and type for CNVR_POP high confidence set. (DOCX)

S3 Table Chromosome, start and end position, size in base pairs (bp), and type for CNVR_ANIMAL high confidence set. (DOCX)

S4 Table Unique high confidence CNVR identification (CNVR), chromosome (BTA), start position, end position, size in base pairs (bp), type (CNVR_POP type, CNVR_ANI type) and number of individuals present in the CNVR (CNVR_POP individuals, CNVR_ANI individuals). (DOCX)

S5 Table Unique high confidence CNVR identification (CNVR), genes and pseudogenes, and QTL and significative (p<0.05) associated traits (QTL and associated traits). (DOCX)

S6 Table MeSH Term Identification (Term ID), description, number of genes (Number) and genes related to significantly enriched MeSH terms (p-adjust<0,05). (DOCX)

S7 Table Identification, chromosome (BTA), start position, and end position of the overlapping CNVR, unique high confidence CNVR identification (CNVR), and type (Type), high confidence CNVR set, DGVa CNVR type and study. (DOCX)
