# Supplementary material for: Identification of copy number variations in the genome of Dairy Gir cattle
Source: PLoS One. 2023 Apr 10;18(4):e0284085. doi: 10.1371/journal.pone.0284085 (PMC10085049; doi:10.1371/journal.pone.0284085)
Supplement: S1 Fig — (A) CNVR_POP set. (B) CNVR_ANI set. (DOCX) [file pone.0284085.s001.docx]

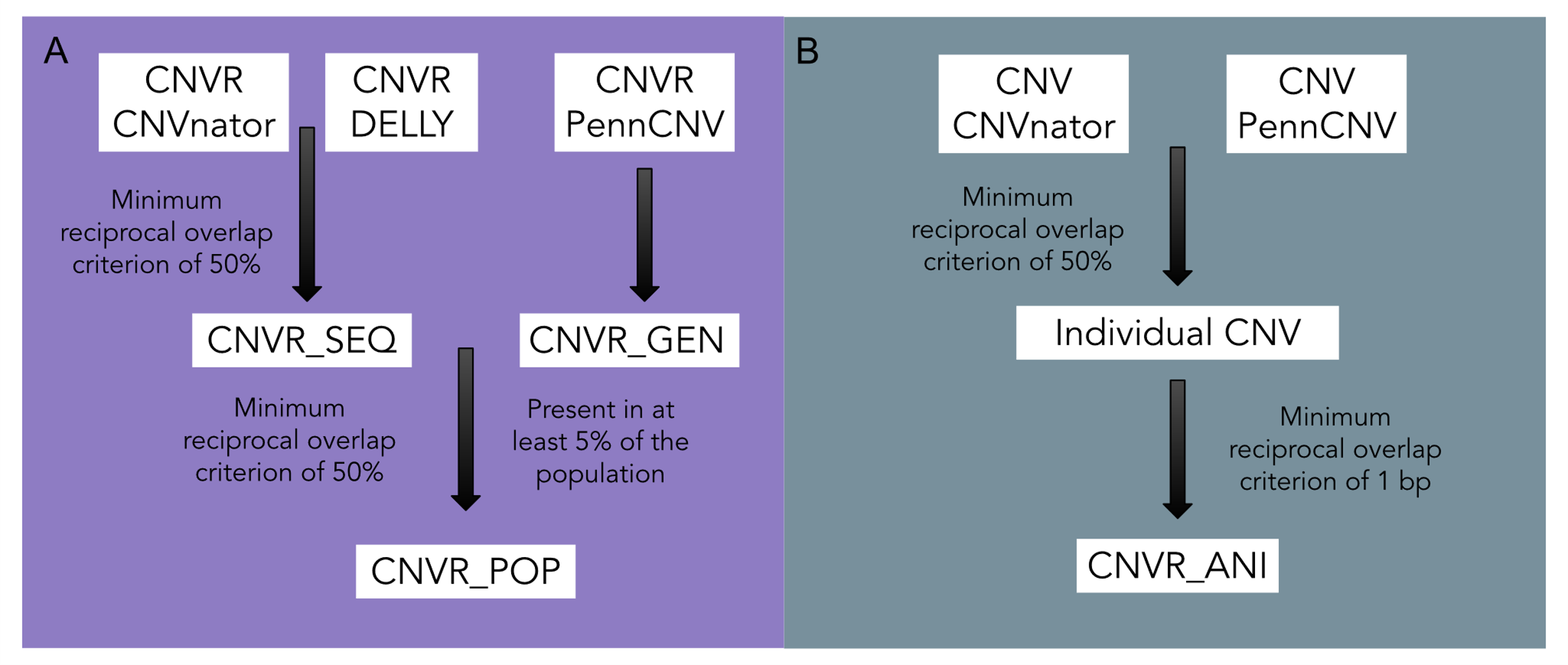


## Fig S1. Definition of the high confidence copy number variation region sets (CNVR). (A) CNVR_POP set. (B) CNVR_ANI set.
