# Supplementary material for: Identification of copy number variations in the genome of Dairy Gir cattle
Source: PLoS One. 2023 Apr 10;18(4):e0284085. doi: 10.1371/journal.pone.0284085 (PMC10085049; doi:10.1371/journal.pone.0284085)
Supplement: S2 Fig — (DOCX) [file pone.0284085.s002.docx]

**
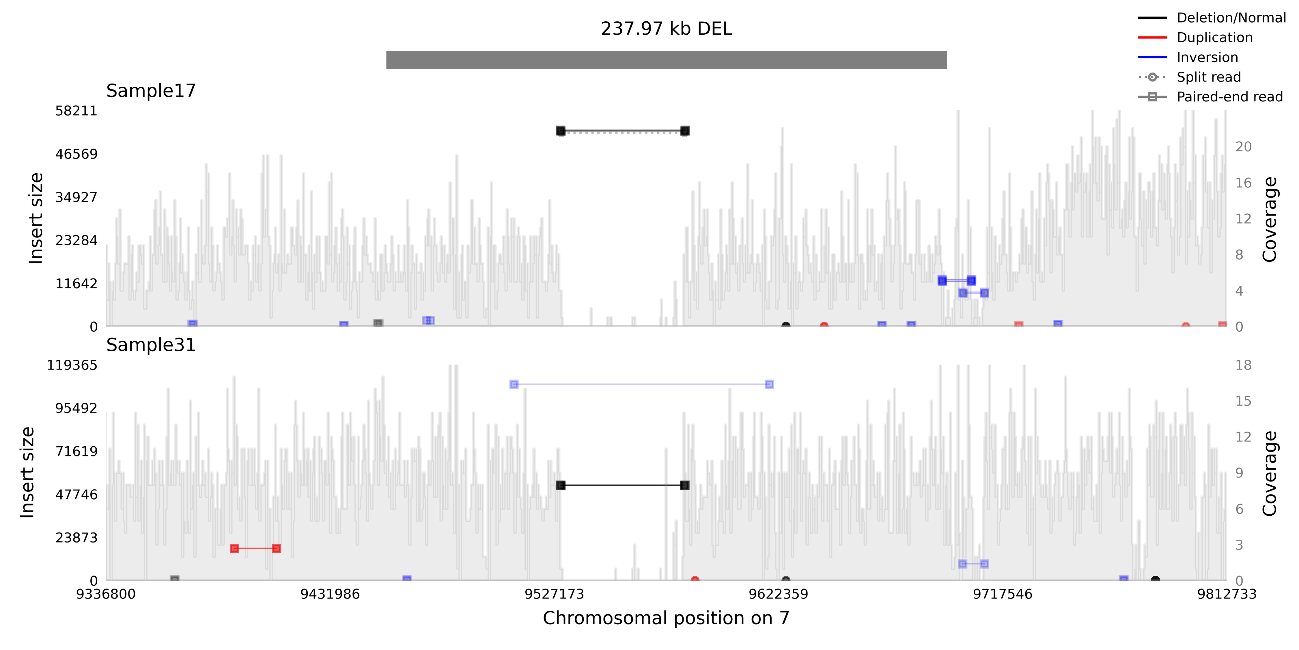
**

**S2 Fig**. Graphical visualization of CNVR14 (BTA7: 9455783-9693750) across different samples showing putative deletion events.
