# Supplementary material for: Identification of copy number variations in the genome of Dairy Gir cattle
Source: PLoS One. 2023 Apr 10;18(4):e0284085. doi: 10.1371/journal.pone.0284085 (PMC10085049; doi:10.1371/journal.pone.0284085)
Supplement: S3 Fig — (DOCX) [file pone.0284085.s003.docx]

**
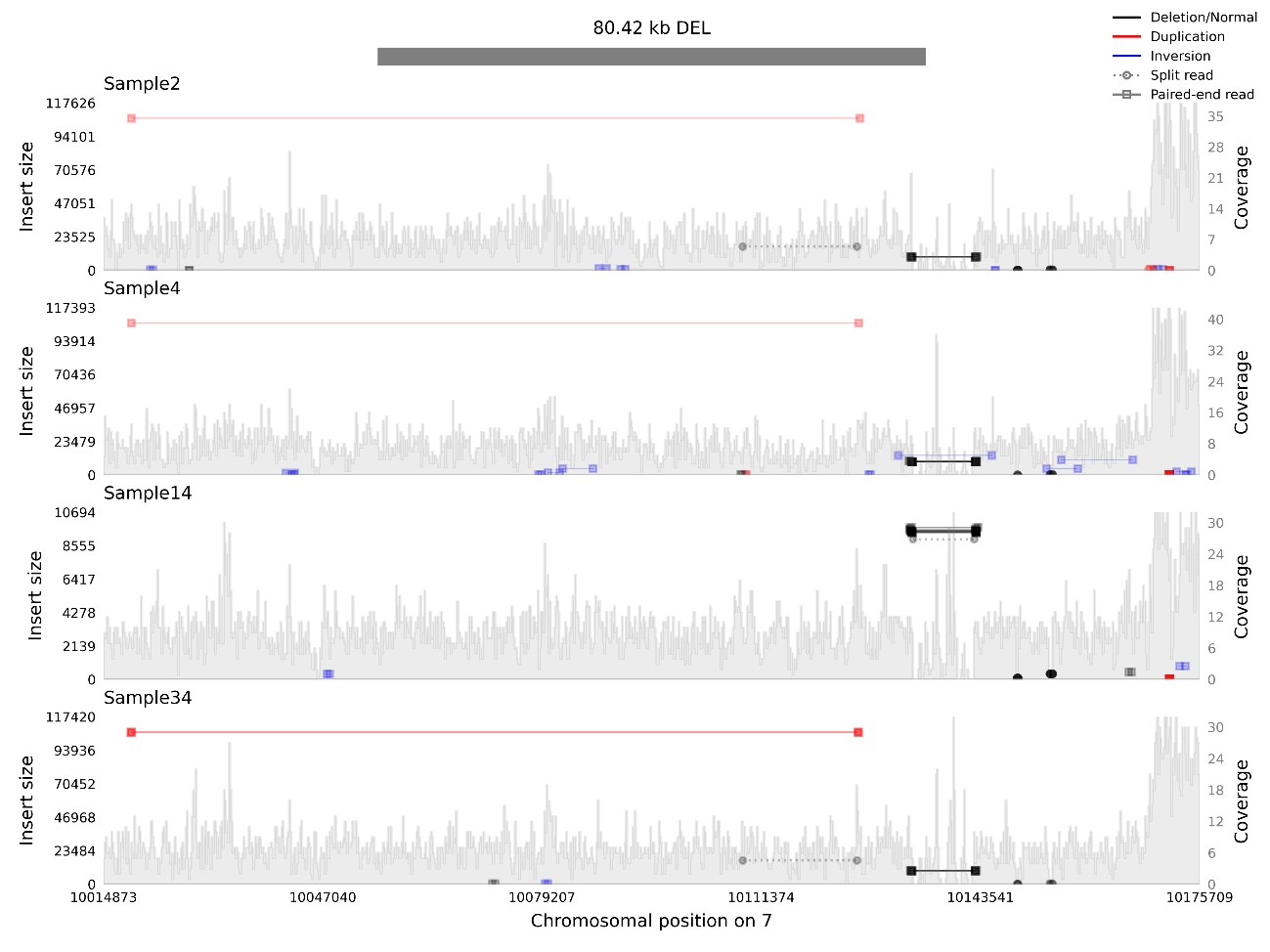
**

**S3 Fig.** Graphical visualization of CNVR16 (BTA7:10055082-10135500) across different samples showing putative deletion events.
