# Supplementary material for: Identification of copy number variations in the genome of Dairy Gir cattle
Source: PLoS One. 2023 Apr 10;18(4):e0284085. doi: 10.1371/journal.pone.0284085 (PMC10085049; doi:10.1371/journal.pone.0284085)
Supplement: S5 Fig — (DOCX) [file pone.0284085.s005.docx]

**
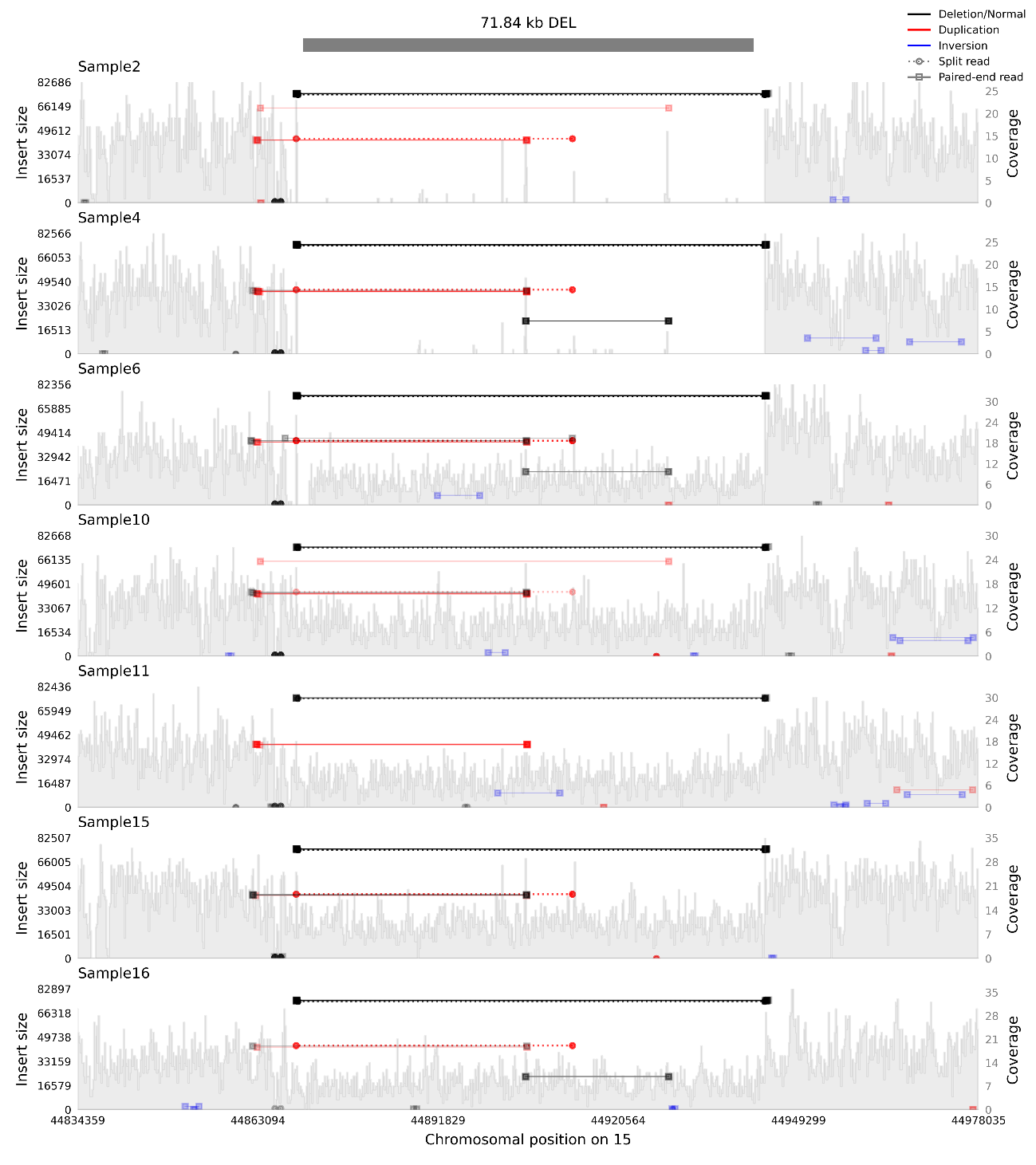
**

**S5 Fig.** Graphical visualization of CNVR34 (BTA15:44870278-44942116) across different samples showing putative deletions events.
