# Supplementary material for: Identification of copy number variations in the genome of Dairy Gir cattle
Source: PLoS One. 2023 Apr 10;18(4):e0284085. doi: 10.1371/journal.pone.0284085 (PMC10085049; doi:10.1371/journal.pone.0284085)
Supplement: S6 Fig — (DOCX) [file pone.0284085.s006.docx]

**
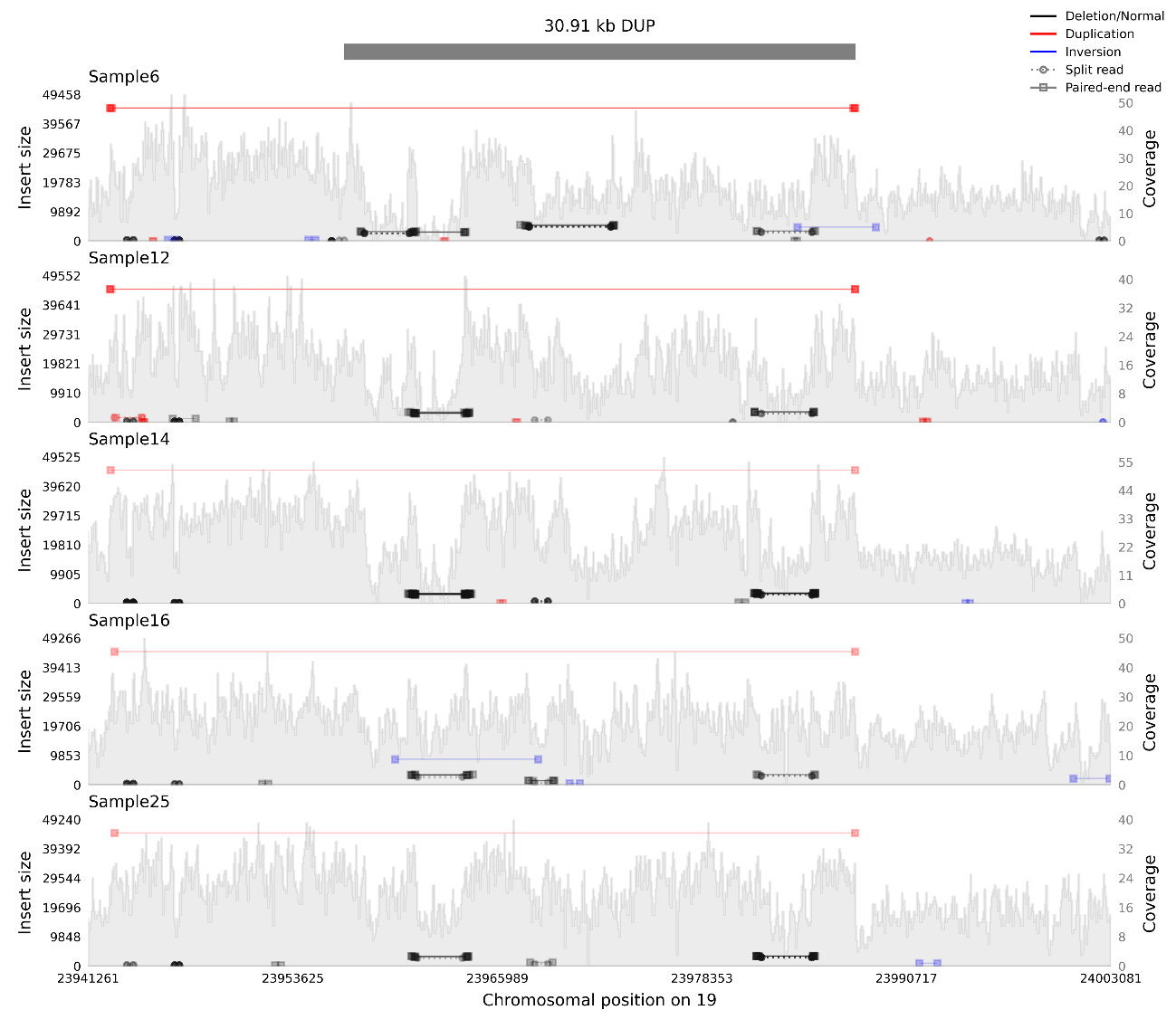
**

**S6 Fig.** Graphical visualization of the CNVR40 (BTA19:23956716-23987626), across different samples showing putative complex events.
