# Supplementary material for: Identification of copy number variations in the genome of Dairy Gir cattle
Source: PLoS One. 2023 Apr 10;18(4):e0284085. doi: 10.1371/journal.pone.0284085 (PMC10085049; doi:10.1371/journal.pone.0284085)
Supplement: S8 Fig — (DOCX) [file pone.0284085.s008.docx]

**
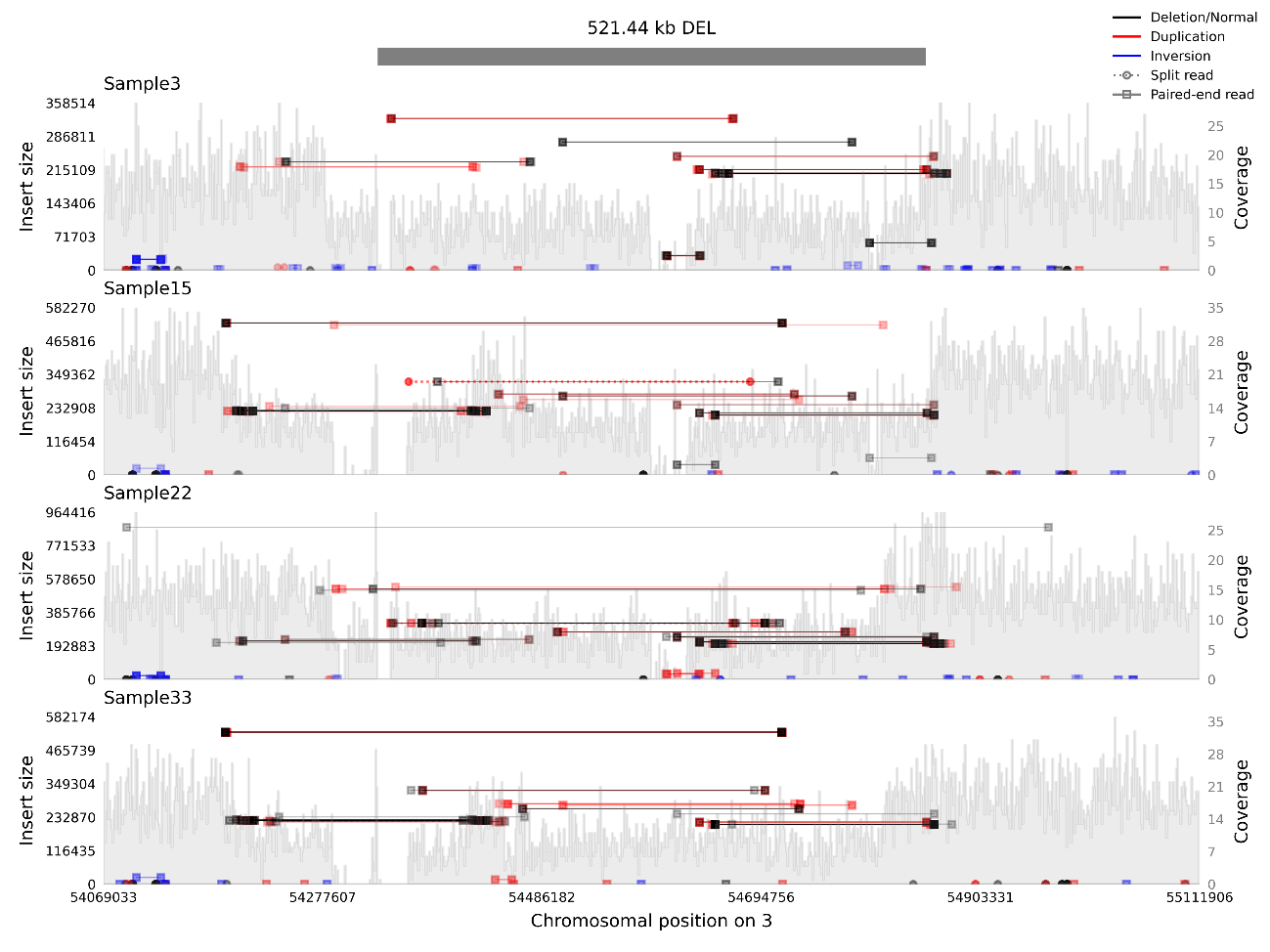
**

**S8 Fig**. Graphical visualization of CNVR8 (BTA3:54329751-54851188) across different samples showing putative deletion events.
