# Supplementary material for: Identification of copy number variations in the genome of Dairy Gir cattle
Source: PLoS One. 2023 Apr 10;18(4):e0284085. doi: 10.1371/journal.pone.0284085 (PMC10085049; doi:10.1371/journal.pone.0284085)
Supplement: S10 Fig — (DOCX) [file pone.0284085.s010.docx]

**
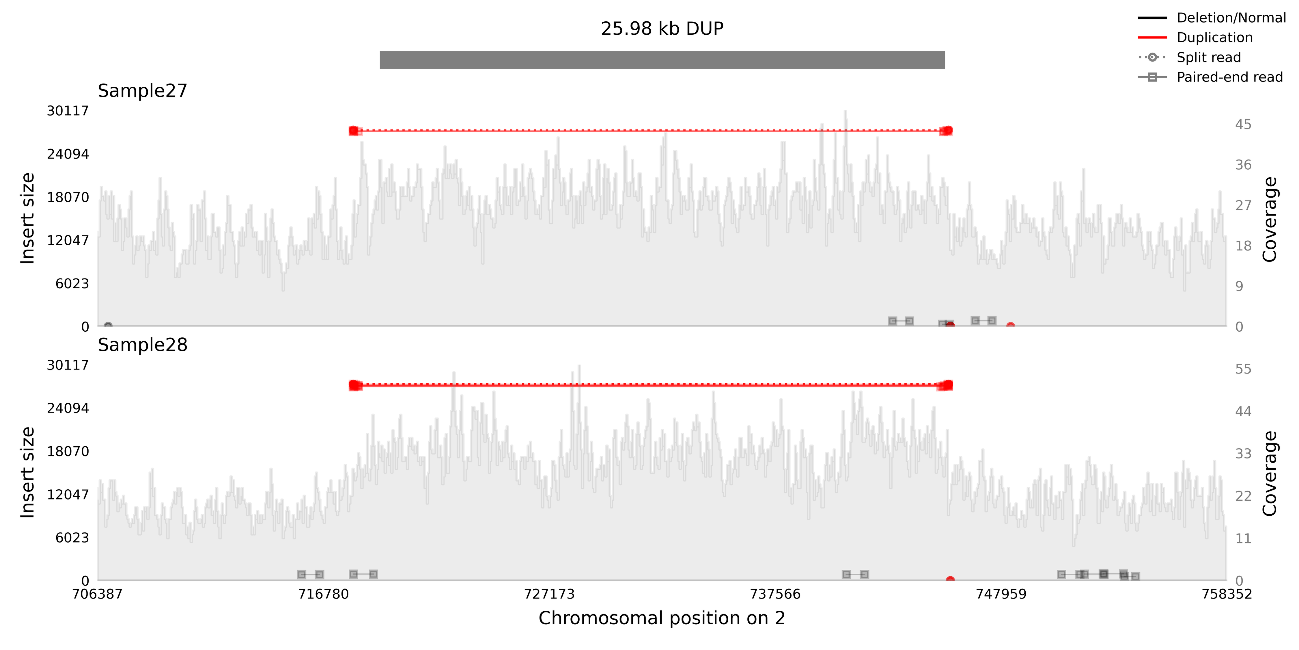
**

**S10 Fig.** Graphical visualization of CNVR3 (BTA2:719378-745361) across different samples showing putative duplication events.
