# Supplementary material for: Identification of copy number variations in the genome of Dairy Gir cattle
Source: PLoS One. 2023 Apr 10;18(4):e0284085. doi: 10.1371/journal.pone.0284085 (PMC10085049; doi:10.1371/journal.pone.0284085)
Supplement: S13 Fig — (DOCX) [file pone.0284085.s013.docx]

**
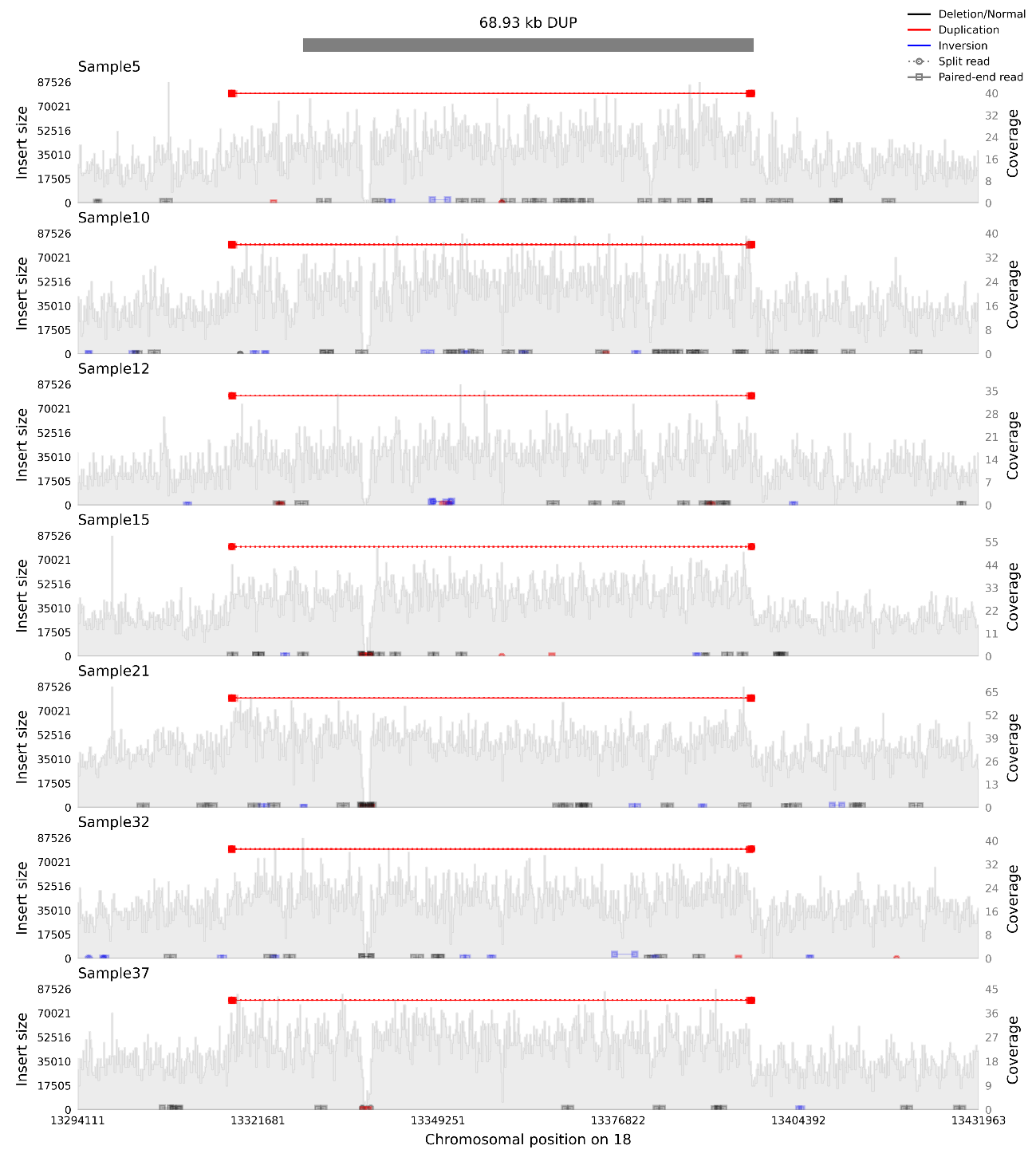
**

**S13 Fig.** Graphical visualization of CNVR37 (BTA18:13328574-13397500) across different samples showing putative duplication events.
