# Supplementary material for: Identification of copy number variations in the genome of Dairy Gir cattle
Source: PLoS One. 2023 Apr 10;18(4):e0284085. doi: 10.1371/journal.pone.0284085 (PMC10085049; doi:10.1371/journal.pone.0284085)
Supplement: S1 Table — (DOCX) [file pone.0284085.s018.docx]

## S1 Table. Sample, total number of reads, percentage of mapped reads (%), percentage of properly paired reads (%), and coverage (X) per sample after duplicates removal.

| Sample | Total number of reads | Mapped reads (%) | Properly paired reads (%) | Coverage (X) |
| --- | --- | --- | --- | --- |
| 1 | 356625327 | 99.72 | 87.27 | 13.6 |
| 2 | 362701796 | 99.79 | 97.82 | 15.0 |
| 3 | 385353621 | 99.81 | 97.48 | 15.9 |
| 4 | 374801680 | 99.80 | 97.48 | 15.6 |
| 5 | 386325249 | 99.85 | 97.54 | 16.0 |
| 6 | 368701320 | 99.77 | 97.97 | 15.2 |
| 7 | 306516816 | 99.79 | 97.56 | 12.6 |
| 8 | 345575226 | 99.79 | 97.04 | 14.0 |
| 9 | 245377907 | 99.75 | 97.49 | 10.2 |
| 10 | 394641626 | 99.80 | 97.70 | 16.1 |
| 11 | 395470276 | 99.83 | 97.96 | 16.2 |
| 12 | 260192208 | 99.64 | 96.74 | 10.3 |
| 13 | 343774440 | 99.87 | 97.79 | 14.8 |
| 14 | 364206965 | 99.79 | 93.89 | 17.7 |
| 15 | 452832085 | 99.81 | 95.98 | 21.5 |
| 16 | 367083050 | 99.72 | 84.38 | 15.6 |
| 17 | 325143647 | 99.79 | 86.33 | 14.1 |
| 18 | 366598393 | 99.87 | 95.76 | 17.7 |
| 19 | 307936476 | 97.79 | 95.76 | 15.2 |
| 20 | 359056254 | 99.70 | 90.39 | 16.7 |
| 21 | 486209902 | 99.78 | 97.17 | 25.0 |
| 22 | 305000681 | 99.66 | 94.08 | 14.8 |
| 23 | 380210484 | 99.78 | 96.15 | 18.8 |
| 24 | 335406862 | 99.71 | 92.29 | 16.2 |
| 25 | 340249508 | 99.79 | 95.49 | 17.0 |
| 26 | 372228475 | 99.64 | 94.32 | 17.1 |
| 27 | 405614167 | 99.87 | 96.80 | 20.1 |
| 28 | 404099122 | 99.72 | 97.80 | 20.9 |
| 29 | 312239045 | 99.63 | 88.37 | 13.9 |
| 30 | 328843430 | 97.56 | 95.54 | 16.7 |
| 31 | 365787842 | 99.79 | 97.30 | 18.8 |
| 32 | 323707726 | 96.59 | 89.11 | 15.0 |
| 33 | 401851858 | 99.81 | 97.98 | 20.3 |
| 34 | 276449451 | 99.80 | 96.56 | 13.9 |
| 35 | 321650759 | 99.85 | 94.35 | 15.8 |
| 36 | 273119499 | 99.79 | 95.04 | 13.6 |
| 37 | 395116189 | 99.78 | 95.81 | 19.3 |
| 38 | 434008561 | 99.88 | 91.13 | 20.3 |
