# Supplementary material for: Identification of copy number variations in the genome of Dairy Gir cattle
Source: PLoS One. 2023 Apr 10;18(4):e0284085. doi: 10.1371/journal.pone.0284085 (PMC10085049; doi:10.1371/journal.pone.0284085)
Supplement: S2 Table — (DOCX) [file pone.0284085.s019.docx]

## S2 Table. Chromosome, start and end position, size in base pairs (bp), and type for CNVR_POP high confidence set

| Chromosome | Start position | End position | Size (bp) | Type |
| --- | --- | --- | --- | --- |
| 2 | 123735242 | 123851299 | 116057 | DELETION |
| 3 | 54329751 | 54851188 | 521437 | COMPLEX |
| 6 | 3202792 | 3240026 | 37234 | DUPLICATION |
| 9 | 5051796 | 5177690 | 125894 | DELETION |
| 9 | 29399118 | 29413997 | 14879 | DELETION |
| 9 | 30698315 | 30726606 | 28291 | DELETION |
| 15 | 44870278 | 44942116 | 71838 | COMPLEX |
| 18 | 13328574 | 13397206 | 68632 | DUPLICATION |
| 19 | 23956716 | 23987626 | 30910 | COMPLEX |
| 26 | 23374431 | 23408689 | 34258 | COMPLEX |
