# Supplementary material for: Identification of copy number variations in the genome of Dairy Gir cattle
Source: PLoS One. 2023 Apr 10;18(4):e0284085. doi: 10.1371/journal.pone.0284085 (PMC10085049; doi:10.1371/journal.pone.0284085)
Supplement: S3 Table — (DOCX) [file pone.0284085.s020.docx]

## S3 Table. Chromosome, start and end position, size in base pairs (bp), and type for CNVR_ANIMAL high confidence set.

| Chromosome | Start position | End position | Size (bp) | Type |
| --- | --- | --- | --- | --- |
| 1 | 18360408 | 18384034 | 23626 | DUPLICATION |
| 1 | 130963070 | 130991359 | 28289 | DUPLICATION |
| 2 | 123765001 | 123851299 | 86298 | DELETION |
| 2 | 719378 | 745361 | 25983 | DUPLICATION |
| 2 | 117790751 | 117904500 | 113749 | DUPLICATION |
| 2 | 134624266 | 134933500 | 309234 | DUPLICATION |
| 3 | 54367531 | 54651000 | 283469 | DELETION |
| 3 | 20917796 | 20944085 | 26289 | DUPLICATION |
| 4 | 82698947 | 82728750 | 29803 | DUPLICATION |
| 4 | 105218001 | 105292500 | 74499 | DUPLICATION |
| 5 | 7733251 | 7765707 | 32456 | DELETION |
| 6 | 11393501 | 11436703 | 43202 | DELETION |
| 7 | 9455783 | 9693750 | 237967 | DELETION |
| 7 | 9739213 | 9793250 | 54037 | DELETION |
| 7 | 10055082 | 10135500 | 80418 | DELETION |
| 7 | 41582849 | 41938000 | 355151 | DELETION |
| 9 | 5054168 | 5177690 | 123522 | DELETION |
| 9 | 29399118 | 29411121 | 12003 | DELETION |
| 9 | 15095199 | 15271750 | 176551 | DUPLICATION |
| 11 | 83535731 | 83559396 | 23665 | DELETION |
| 11 | 26400251 | 26444703 | 44452 | DUPLICATION |
| 12 | 59242099 | 59433070 | 190971 | DELETION |
| 12 | 70538501 | 70738500 | 199999 | DELETION |
| 12 | 71894273 | 71953261 | 58988 | DELETION |
| 12 | 167702 | 262500 | 94798 | DUPLICATION |
| 12 | 71187501 | 71259000 | 71499 | DUPLICATION |
| 12 | 71334251 | 71418750 | 84499 | DUPLICATION |
| 13 | 2199336 | 2238554 | 39218 | DELETION |
| 13 | 53461848 | 53511604 | 49756 | DELETION |
| 13 | 12487232 | 12761250 | 274018 | DUPLICATION |
| 14 | 79478001 | 79499712 | 21711 | DUPLICATION |
| 15 | 44881987 | 44933173 | 51186 | DELETION |
| 16 | 32607925 | 32655581 | 47656 | DELETION |
| 17 | 26898751 | 26929822 | 31071 | DELETION |
| 18 | 13344360 | 13397500 | 53140 | DUPLICATION |
| 18 | 58916664 | 59054123 | 137459 | DUPLICATION |
| 18 | 64384251 | 64406577 | 22326 | DUPLICATION |
| 20 | 3549957 | 3609244 | 59287 | DELETION |
| 20 | 57454844 | 57467750 | 12906 | DELETION |
| 21 | 58680616 | 58696778 | 16162 | DUPLICATION |
| 23 | 25679501 | 25705975 | 26474 | DELETION |
| 26 | 23378751 | 23408689 | 29938 | DUPLICATION |
| 28 | 123251 | 413750 | 290499 | DELETION |
| 28 | 627488 | 934000 | 306512 | DUPLICATION |
| 28 | 6398983 | 6451134 | 52151 | DUPLICATION |
