# Supplementary material for: Identification of copy number variations in the genome of Dairy Gir cattle
Source: PLoS One. 2023 Apr 10;18(4):e0284085. doi: 10.1371/journal.pone.0284085 (PMC10085049; doi:10.1371/journal.pone.0284085)
Supplement: S4 Table — (DOCX) [file pone.0284085.s021.docx]

## S4 Table. Unique high confidence CNVR identification (CNVR), chromosome (BTA), start position, end position, size in base pairs (bp), type (CNVR_POP type, CNVR_ANI type) and number of individuals present in the CNVR (CNVR_POP individuals, CNVR_ANI individuals).

| CNVR | BTA | Start position (bp) | End position (bp) | Size (bp) | CNVR_POP type | CNVR_ANI type | CNVR_POP* individuals | | CNVR_ANI individuals |
| --- | --- | --- | --- | --- | --- | --- | --- | --- | --- |
| CNVR1 | 1 | 18360408 | 18384034 | 23626 | - | DUPLICATION | - | 5 | |
| CNVR2 | 1 | 130963070 | 130991359 | 28289 | - | DUPLICATION | - | 1 | |
| CNVR3 | 2 | 719378 | 745361 | 25983 | - | DUPLICATION | - | 2 | |
| CNVR4 | 2 | 117790751 | 117904500 | 113749 | - | DUPLICATION | - | 2 | |
| CNVR5 | 2 | 123735242 | 123851299 | 116057 | DELETION | DELETION | 51 (9.32%) | 3 | |
| CNVR6 | 2 | 134624266 | 134933500 | 309234 | - | DUPLICATION |  | 2 | |
| CNVR7 | 3 | 20917796 | 20944085 | 26289 | - | DUPLICATION |  | 1 | |
| CNVR8 | 3 | 54329751 | 54851188 | 521437 | COMPLEX | DELETION | 185 (33.82%) | 3 | |
| CNVR9 | 4 | 82698947 | 82728750 | 29803 | - | DUPLICATION | - | 10 | |
| CNVR10 | 4 | 105218001 | 105292500 | 74499 | - | DUPLICATION | - | 1 | |
| CNVR11 | 5 | 7733251 | 7765707 | 32456 | - | DELETION | - | 1 | |
| CNVR12 | 6 | 3202792 | 3240026 | 37234 | DUPLICATION | - | 28 (5.12%) | - | |
| CNVR13 | 6 | 11393501 | 11436703 | 43202 | - | DELETION |  | 3 | |
| CNVR14 | 7 | 9455783 | 9693750 | 237967 | - | DELETION | - | 2 | |
| CNVR15 | 7 | 9739213 | 9793250 | 54037 | - | DELETION | - | 4 | |
| CNVR16 | 7 | 10055082 | 10135500 | 80418 | - | DELETION | - | 4 | |
| CNVR17 | 7 | 41582849 | 41938000 | 355151 | - | DELETION | - | 6 | |
| CNVR18 | 9 | 5051796 | 5177690 | 125894 | DELETION | DELETION | 32 (5.85%) | 12 | |
| CNVR19 | 9 | 15095199 | 15271750 | 176551 | - | DUPLICATION | - | 13 | |
| CNVR20 | 9 | 29399118 | 29413997 | 14879 | DELETION | DELETION | 72 (13.16%) | 2 | |
| CNVR21 | 9 | 30698315 | 30726606 | 28291 | DELETION | **-** | 40 (73.13%) | - | |
| CNVR22 | 11 | 26400251 | 26444703 | 44452 | - | DUPLICATION | - | 7 | |
| CNVR23 | 11 | 83535731 | 83559396 | 23665 | - | DELETION | - | 1 | |
| CNVR24 | 12 | 167702 | 262500 | 94798 | - | DUPLICATION | - | 1 | |
| CNVR25 | 12 | 59242099 | 59433070 | 190971 | - | DELETION | - | 2 | |
| CNVR26 | 12 | 70538501 | 70738500 | 199999 | - | DELETION | - | 1 | |
| CNVR27 | 12 | 71187501 | 71259000 | 71499 | - | DUPLICATION | - | 2 | |
| CNVR28 | 12 | 71334251 | 71418750 | 84499 | - | DUPLICATION | - | 3 | |
| CNVR29 | 12 | 71894273 | 71953261 | 58988 | - | DELETION | - | 4 | |
| CNVR30 | 13 | 2199336 | 2238554 | 39218 | - | DELETION | - | 1 | |
| CNVR31 | 13 | 12487232 | 12761250 | 274018 | - | DUPLICATION | - | 13 | |
| CNVR32 | 13 | 53461848 | 53511604 | 49756 | - | DELETION | - | 14 | |
| CNVR33 | 14 | 79478001 | 79499712 | 21711 | - | DUPLICATION | - | 2 | |
| CNVR34 | 15 | 44870278 | 44942116 | 71838 | COMPLEX | DELETION | 68 (12.43%) | 1 | |
| CNVR35 | 16 | 32607925 | 32655581 | 47656 | - | DELETION | - | 1 | |
| CNVR36 | 17 | 26898751 | 26929822 | 31071 | - | DELETION | - | 11 | |
| CNVR37 | 18 | 13328574 | 13397500 | 68926 | DUPLICATION | DUPLICATION | 98 (17.91%) | 1 | |
| CNVR38 | 18 | 58916664 | 59054123 | 137459 | - | DUPLICATION | - | 1 | |
| CNVR39 | 18 | 64384251 | 64406577 | 22326 | - | DUPLICATION | - | 2 | |
| CNVR40 | 19 | 23956716 | 23987626 | 30910 | COMPLEX | - | 39 (7.13%) | - | |
| CNVR41 | 20 | 3549957 | 3609244 | 59287 | - | DELETION | - | 1 | |
| CNVR42 | 20 | 57454844 | 57467750 | 12906 | - | DELETION | - | 3 | |
| CNVR43 | 21 | 58680616 | 58696778 | 16162 | - | DUPLICATION | - | 1 | |
| CNVR44 | 23 | 25679501 | 25705975 | 26474 | - | DELETION | - | 1 | |
| CNVR45 | 26 | 23374431 | 23408689 | 34258 | COMPLEX | DUPLICATION | 36 (6.58%) | 3 | |
| CNVR46 | 28 | 123251 | 413750 | 290499 | - | DELETION | - | 6 | |
| CNVR47 | 28 | 627488 | 934000 | 306512 | - | DUPLICATION | - | 1 | |
| CNVR48 | 28 | 6398983 | 6451134 | 52151 | - | DUPLICATION | - | 1 | |

* Relative frequency is shown between parentheses
