# Supplementary material for: Identification of copy number variations in the genome of Dairy Gir cattle
Source: PLoS One. 2023 Apr 10;18(4):e0284085. doi: 10.1371/journal.pone.0284085 (PMC10085049; doi:10.1371/journal.pone.0284085)
Supplement: S5 Table — (DOCX) [file pone.0284085.s022.docx]

## S5 Table. Unique high confidence CNVR identification (CNVR), genes and pseudogenes, and QTL and significative (p<0.05) associated traits (QTL and associated traits).

| CNVR | Genes and pseudogenes | QTL and associated traits* |
| --- | --- | --- |
| 1 | - | - |
| 2 | - | - |
| 3 | *HERC2* | - |
| 4 | *ENSBTAG00000049805, TRIP12* | - |
| 5 | - | - |
| 6 | *ACTL8* | Production (66904) - Body weight gain; Milk (125440) - Lactation persistency |
| 7 | *MGC134040* | - |
| 8 | *ENSBTAG00000002416, GBP4, ENSBTAG00000024272, GBP6, ENSBTAG00000038233, GBP2, U2* | Reproduction (181182, 181446) - Conception rate, Inseminations per conception |
| 9 | *ENSBTAG00000002859, ENSBTAG00000055254* | - |
| 10 | *CLEC5A, TAS2R38, MGAM* | - |
| 11 | - | - |
| 12 | - | - |
| 13 | - | - |
| 14 | *OR7A95, ENSBTAG00000050759, OR7A78, OR7A99, OR7A97, ENSBTAG00000054398* | - |
| 15 | *ENSBTAG00000047589* | - |
| 16 | *OR7A112* | - |
| 17 | *OR2M16, OR2L13, OR2AJ9, OR2T22, OR2AJ10P* (pseudogene)*, OR2L2C, OR2L2B, OR2L2, OR2L3C* | Milk (64049, 64050) - Milk riboflavin content |
| 18 | - | - |
| 19 | *FILIP1, SENP6, ENSBTAG00000032382* | Health (167889) - Bovine tuberculosis susceptibility |
| 20 | - | - |
| 21 | - | - |
| 22 | *ENSBTAG00000016794, U6, ENSBTAG00000054517* | - |
| 23 | - | Production (45767, 45772, 45775, 45780) – Body depth, PTA type, Net merit, Rump width; Reproduction (45768, 45781) -Calving ease (maternal), Calving ease; Conformação (45769, 45770, 45773, 45778, 45782, 45783) - Foot angle, Feet and leg conformation, Udder attachment, Rear leg placement - rear view, Stature, Strength |
| 24 | - | - |
| 25 | - | - |
| 26 | *ENSBTAG00000046041* | - |
| 27 | *ENSBTAG00000049836* | - |
| 28 | *ENSBTAG00000026070* | Health (211939) - M. paratuberculosis susceptibility |
| 29 | *ENSBTAG00000052990* | - |
| 30 | *ENSBTAG00000054174* | - |
| 31 | *ECHDC3, USP6NL* | Meat and Carcass (36961) – Lean meat yield |
| 32 | *SIRPB1, ENSBTAG00000054594* | - |
| 33 | *-* | - |
| 34 | *OR10AB6, OR10AB2, OR5P90P* (pseudogene)*, OR5P1C, OR5P76B* | - |
| 35 | ENSBTAG00000044066 | - |
| 36 | - | - |
| 37 | *CA5A, BANP* | - |
| 38 | *ENSBTAG00000015899, ENSBTAG00000052265, ENSBTAG00000054310* | Production (123784, 123053, 123250, 123656, 122886, 122887, 123291) – Length of productive life; Reproduction (147014, 147022) – Calving ease (maternal), Calving ability |
| 39 | *ENSBTAG00000050946* | - |
| 40 | *OR1P1, U6* | - |
| 41 | - | Reproduction (139139) – Scrotal circumference; Milk (158256) – Milking speed |
| 42 | - | Health (179855, 179913) - Ketosis |
| 43 | *ENSBTAG00000031834* | Exterior (125875, 125954) – Feet and leg conformation, Bone quality |
| 44 | *BLA-DQB, ENSBTAG00000037605 (DQA1)* | Health (153931) – Bovine leukemia virus susceptibility |
| 45 | *WBP1L* | - |
| 46 | *OR5AS1, OR5D18K, OR5L20* | - |
| 47 | *RHOU* | Health (179050) - Bovine tuberculosis susceptibility; Reproduction (53666, 53667, 53673) – Calving ease (maternal), Stillbirth (maternal), Calving ease; Production (53670) – Net merit |
| 48 | - | Milk (173141, 173244) – Milk lactose content, Milk mid-infrared spectra |

* QTL identification is shown between parenthesis.
