# Supplementary material for: Identification of copy number variations in the genome of Dairy Gir cattle
Source: PLoS One. 2023 Apr 10;18(4):e0284085. doi: 10.1371/journal.pone.0284085 (PMC10085049; doi:10.1371/journal.pone.0284085)
Supplement: S6 Table — (DOCX) [file pone.0284085.s023.docx]

## S6 Table. MeSH Term Identification (Term ID), description, number of genes (Number) and genes related to significantly enriched MeSH terms (p-adjust<0,05).

| Term ID | Description | Number | Genes |
| --- | --- | --- | --- |
| Anatomy (A) | | | |
| D015496 | CD4-Positive T-Lymphocytes | 2 | *BoLA-DQB, BOLA-DQA1* |
| Chemicals and Drugs (D) | | | |
| D001425 | Bacterial Outer Membrane Proteins | 2 | *BoLA-DQB, BOLA-DQA1* |
| D000949 | Histocompatibility Antigens Class II | 2 | *BoLA-DQB, BOLA-DQA1* |
| D000953 | Antigens, Protozoan | 2 | *BoLA-DQB, BOLA-DQA1* |
| D018122 | B7-1 Antigen | 2 | *BoLA-DQB, BOLA-DQA1* |
| D051940 | B7-2 Antigen | 2 | *BoLA-DQB, BOLA-DQA1* |
| D006683 | HLA-DQ Antigens | 2 | *BoLA-DQB, BOLA-DQA1* |
| D006684 | HLA-DR Antigens | 1 | *BoLA-DQB* |
| D059848 | HLA-DQ alpha-Chains | 1 | *BOLA-DQA1* |
| D059866 | HLA-DQ beta-Chains | 1 | *BoLA-DQB* |
| D000911 | Antibodies, Monoclonal | 1 | *BoLA-DQB* |
| D021382 | Protein Sorting Signals | 1 | *BoLA-DQB* |
| D000939 | Epitopes | 1 | *BoLA-DQB* |
| D019204 | GTP-Binding Proteins | 1 | *GBP4* |
| Biological Sciences (G) | | | |
| D017951 | Antigen Presentation | 2 | *BoLA-DQB, BOLA-DQA1* |
| D020131 | Genes, Duplicate | 1 | *BoLA-DQB* |
| D056915 | DNA Copy Number Variations | 1 | *GBP4* |
