# Supplementary material for: Identification of copy number variations in the genome of Dairy Gir cattle
Source: PLoS One. 2023 Apr 10;18(4):e0284085. doi: 10.1371/journal.pone.0284085 (PMC10085049; doi:10.1371/journal.pone.0284085)
Supplement: S7 Table — (DOCX) [file pone.0284085.s024.docx]

## S7 Table. Identification, chromosome (BTA), start position, and end position of the overlapping CNVR, unique high confidence CNVR identification (CNVR), and type (Type), high confidence CNVR set, DGVa CNVR type and study.

| BTA | Start position | End position | CNVR | Type | High confidence CNVR set | DGVa CNVR type | DGVa CNVR Study |
| --- | --- | --- | --- | --- | --- | --- | --- |
| 2 | 123735242 | 123802863 | CNVR5 | DELETION | CNVR_POP, CNVR_ANI | COMPLEX, COPY NUMBER VARIATION | Hou et al., 2012, Karimi et al., 2016 |
| 3 | 54329751 | 54851188 | CNVR8 | COMPLEX | CNVR_POP, CNVR_ANI | COMPLEX, COPY NUMBER VARIATION | Hou et al., 2011, Hou et al., 2012, Boussaha et al., 2015, Bickhart et al., 2012, Mesbah-Uddin et al., 2018, Keel et al., 2016, Liu et al., 2010, Karimi et al., 2016 |
| 4 | 105218001 | 105292500 | CNVR10 | DUPLICATION | CNVR_ANI | COMPLEX, COPY NUMBER VARIATION | Bickhart et al., 2012, Boussaha et al., 2015, Liu et al., 2010, Mesbah-Uddin et al., 2018 |
| 7 | 41582849 | 41938000 | CNVR17 | DELETION | CNVR_ANI | COMPLEX, COPY NUMBER VARIATION | Boussaha et al., 2015, Hou et al., 2011, Keel et al., 2016, Mesbah-Uddin et al., 2018, Karimi et al., 2016, Bickhart et al., 2012 |
| 9 | 15095199 | 15264009 | CNVR19 | DUPLICATION | CNVR_ANI | COMPLEX, COPY NUMBER VARIATION | Karimi et al., 2016, Hou et al., 2011, Mesbah-Uddin et al., 2018 |
| 9 | 30698407 | 30714602 | CNVR21 | DELETION | CNVR_POP | DELETION, COPY NUMBER VARIATION | Mesbah-Uddin et al., 2018, Keel et al., 2016 |
| 12 | 59242099 | 59417457 | CNVR25 | DELETION | CNVR_ANI | DELETION, COPY NUMBER VARIATION | Mesbah-Uddin et al., 2018, Hou et al., 2011 |
| 28 | 244723 | 413750 | CNVR46 | DELETION | CNVR_ANI | DELETION, COPY NUMBER VARIATION | Keel et al., 2016, Karimi et al., 2016, Mesbah-Uddin et al., 2018 |
